# Duodenal Laparoscopy and Endoscopy Cooperative Surgery Combined With Endoscopic Nasopancreatic Drainage and Endoscopic Nasobiliary Drainage for a Duodenal Adenoma Near the Papilla of Vater: A Case Report

**DOI:** 10.1002/deo2.70390

**Published:** 2026-09-03

**Authors:** Keisuke Manaka, Hiroki Harada, Takuya Wada, Gen Kitahara, Kosuke Okuwaki, Kota Okuno, Shohei Fujita, Tadashi Higuchi, Koshi Kumagai, Naoki Hiki

**Affiliations:** ^1^ Department of Upper Gastrointestinal Surgery Kitasato University School of Medicine Kanagawa Japan; ^2^ Department of Gastroenterology Kitasato University School of Medicine Kanagawa Japan

**Keywords:** duodenal adenoma, duodenal laparoscopy and endoscopy cooperative surgery, endoscopic nasobiliary drainage, endoscopic nasopancreatic drainage, papilla of Vater

## Abstract

Duodenal laparoscopy and endoscopy cooperative surgery (D‐LECS) for superficial non‐ampullary duodenal tumors has been increasingly performed as a minimally invasive treatment for lesions with a high risk of complications when treated by endoscopy alone. However, for tumors near the papilla of Vater, the optimal treatment strategy has not been established because of the risk of pancreatitis and cholangitis associated with papillary injury. We report a case of duodenal adenoma near the papilla of Vater successfully treated by D‐LECS combined with endoscopic nasopancreatic drainage (ENPD) and endoscopic nasobiliary drainage (ENBD). A 70‐year‐old woman was found to have a 0‐IIa adenoma adjacent to the papilla in the descending portion of the duodenum and underwent D‐LECS with endoscopic retrograde cholangiopancreatography assistance. After Kocher mobilization, endoscopic en bloc resection was performed. The mucosal defect was closed using endoscopic mucosal closure and reinforced with laparoscopic seromuscular suturing. To prevent postoperative complications, ENPD and ENBD were performed. Pathological examination confirmed negative resection margins, and the patient was discharged with an uneventful postoperative course. ENPD and ENBD after D‐LECS were technically feasible without interfering with endoscopic manipulation. This combined approach may be a selective organ‐preserving strategy for carefully selected duodenal tumors located near the papilla of Vater.

## 1 Introduction

Superficial non‐ampullary duodenal epithelial tumors are increasingly detected with the widespread use of upper gastrointestinal endoscopy.

Endoscopic mucosal resection (EMR) is effective for small lesions; however, larger lesions often require piecemeal resection, compromising histopathological evaluation and increasing the risk of recurrence [1].

Endoscopic submucosal dissection (ESD) enables en bloc resection, but ESD in the duodenum remains technically challenging because of the thin duodenal wall and narrow lumen, resulting in a high risk of perforation and delayed adverse events [2, 3].

Duodenal laparoscopy and endoscopy cooperative surgery (D‐LECS) has been developed as a minimally invasive procedure combining ESD with laparoscopic reinforcement to improve safety while preserving organ function [5, 6]. However, for lesions near the papilla of Vater, postoperative edema or thermal injury around the papilla may impair pancreaticobiliary outflow, and the optimal preventive strategy remains unclear. Here, we report a case of duodenal adenoma near the papilla of Vater successfully treated by D‐LECS using the reopenable clip‐over‐the‐line method (ROLM) combined with prophylactic endoscopic nasopancreatic drainage (ENPD) and endoscopic nasobiliary drainage (ENBD).

## 2 Case Report

A 70‐year‐old woman was referred to our hospital after a duodenal lesion was detected during screening upper gastrointestinal endoscopy. Her past medical history included chronic kidney disease, Hashimoto's disease, and systemic sclerosis. She had no history of abdominal surgery.

**FIGURE 1:**
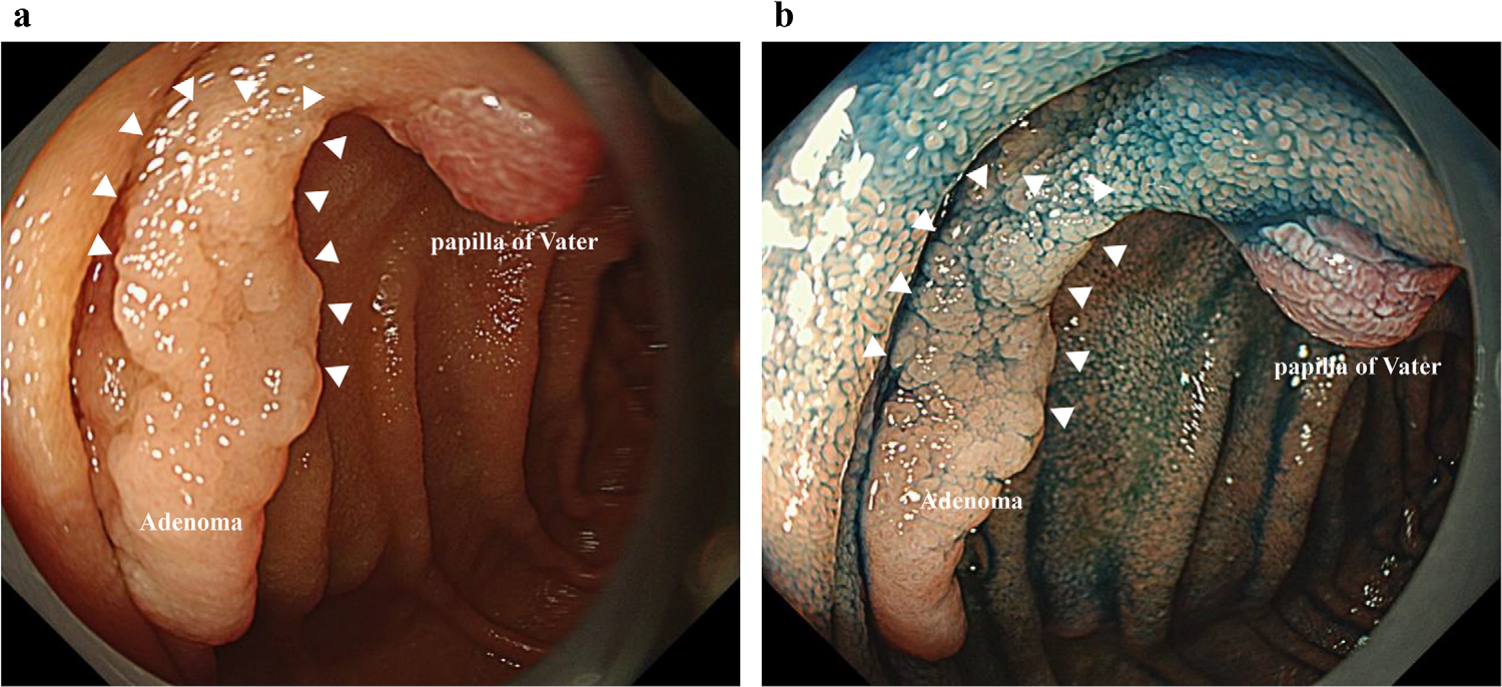
Endoscopic findings. (a) Conventional white‐light endoscopy showing the relationship between the tumor and the papilla of Vater. (b) Endoscopic view after marking and indigo carmine spraying. Both images demonstrate the close proximity between the tumor and the papilla of Vater. Arrowheads indicate the outline of the tumor.

Upper gastrointestinal endoscopy revealed a slightly elevated, well‐demarcated, whitish lesion measuring approximately 10 mm, located in the descending portion of the duodenum near the papilla of Vater (Figure 1a,b). The closest distance between the tumor margin and the papilla of Vater was approximately 3 mm. The lesion did not involve the papillary orifice or the papillary mound, although it was located in very close proximity to them. Biopsy demonstrated an adenoma. Conventional endoscopy showed no findings suggestive of deep submucosal invasion, such as depression, ulceration, or fold convergence. Therefore, the lesion was considered to be confined to the mucosal layer, and endoscopic ultrasonography was not performed. Because of its proximity to the papilla, ESD alone was considered technically demanding, and post‐resection defect management was expected to be difficult. Therefore, D‐LECS was selected to improve endoscopic operability through Kocher mobilization while allowing intraperitoneal assessment and additional reinforcement after resection. After multidisciplinary discussion, D‐LECS with subsequent ERCP and prophylactic ENPD/ENBD was planned.

**FIGURE 2:**
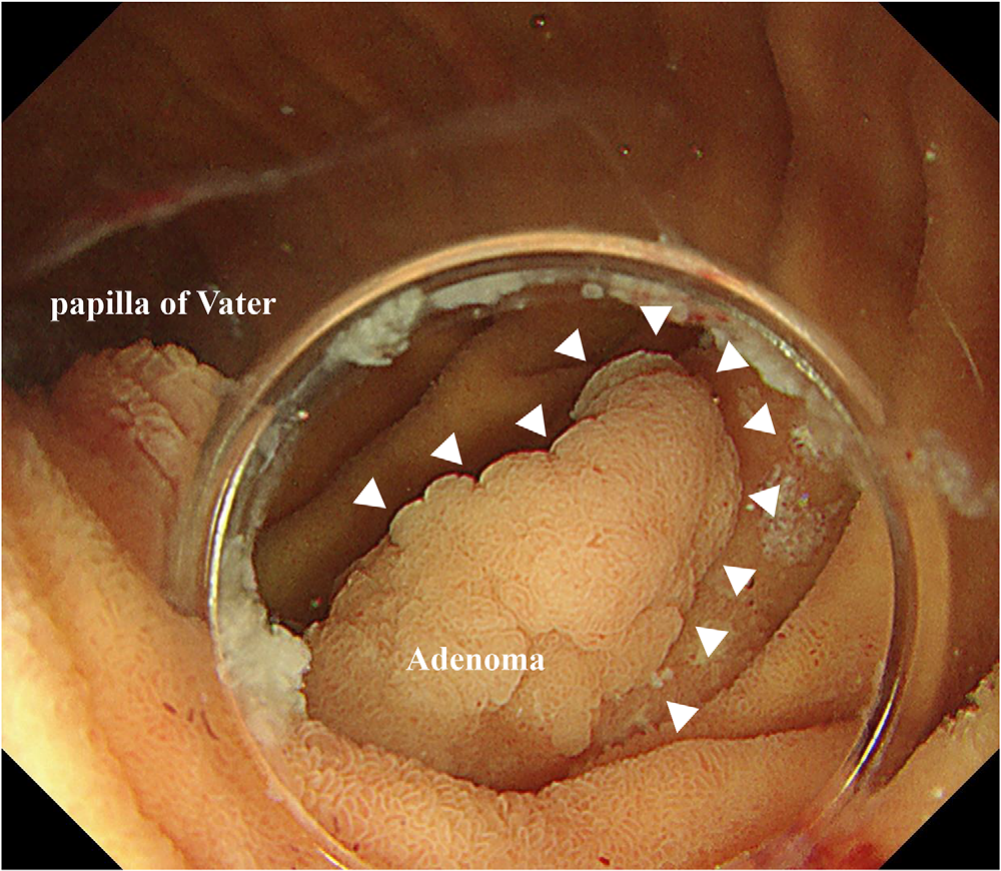
Endoscopic findings after Kocher mobilization. Endoscopic view after Kocher mobilization. Release of retroperitoneal fixation resulted in a more linear endoscopic approach, improving scope stability and facilitating precise endoscopic manipulation.

**FIGURE 3:**
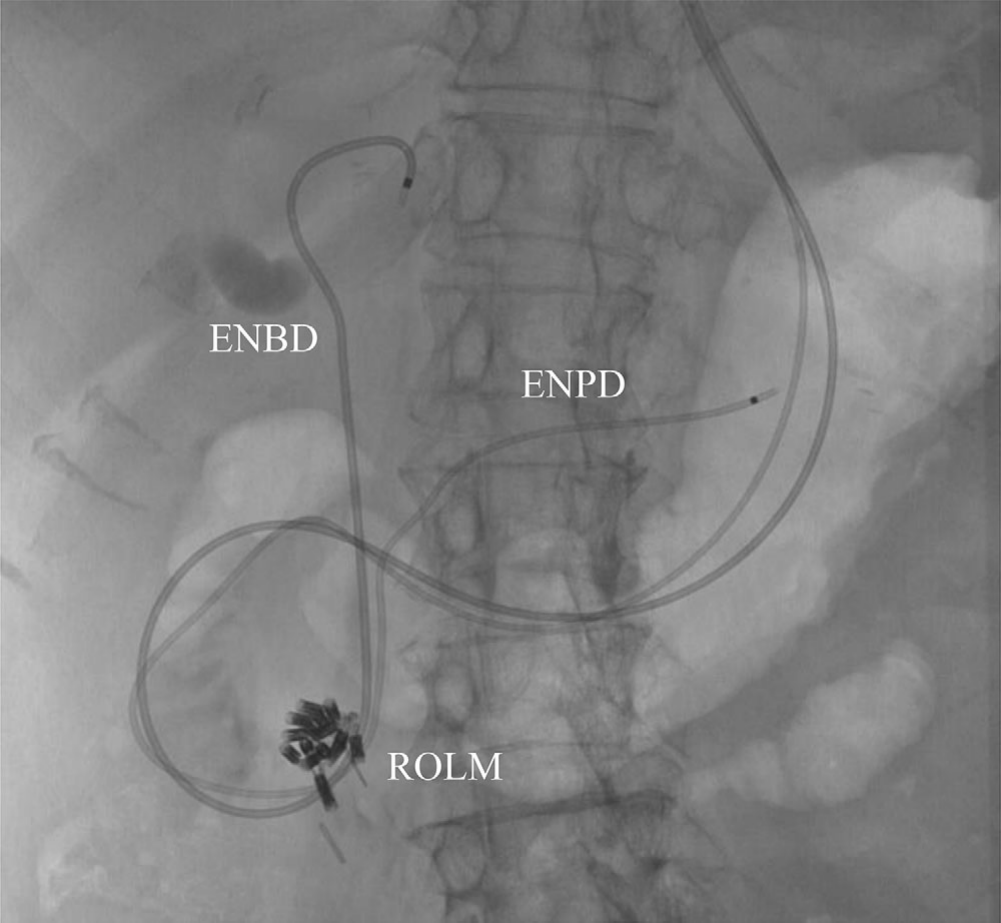
Fluoroscopic findings during endoscopic retrograde cholangiopancreatography. The courses of the endoscopic nasopancreatic drainage (ENPD) and endoscopic nasobiliary drainage (ENBD) tubes are visible, along with the clips used for the reopenable clip‐over‐the‐line method (ROLM).

The operation was performed under general anesthesia using five ports. After opening the omental bursa, the hepatic flexure of the colon was mobilized, followed by the Kocher maneuver to mobilize and straighten the duodenum. The lesion was then resected en bloc by ESD. The mucosal defect was closed endoscopically using the ROLM, and additional reinforcement was performed by laparoscopic seromuscular suturing. Although the lesion could be identified before Kocher mobilization, scope stability was suboptimal. After Kocher mobilization, the endoscopic approach became more linear, improving scope stability and facilitating ESD and subsequent defect closure (Figure 2). Because the lesion was located near the papilla of Vater, postoperative edema or thermal injury could impair pancreaticobiliary outflow, potentially resulting in pancreatitis or cholangitis. ENPD and ENBD were performed under general anesthesia after D‐LECS. ENPD and ENBD were placed only after completion of tumor resection and defect closure, avoiding interference with endoscopic manipulation (Figure 3). After ENPD and ENBD placement, laparoscopic observation confirmed the closure site, followed by retroperitoneal fixation of the duodenum and drain placement (Video S1).

The total procedural time was 356 min, with a blood loss of 50 mL (Supporting Information S1). ENPD and ENBD were removed on postoperative day 5, oral intake was resumed, and the patient was discharged on postoperative day 13 without complications.

**FIGURE 4:**
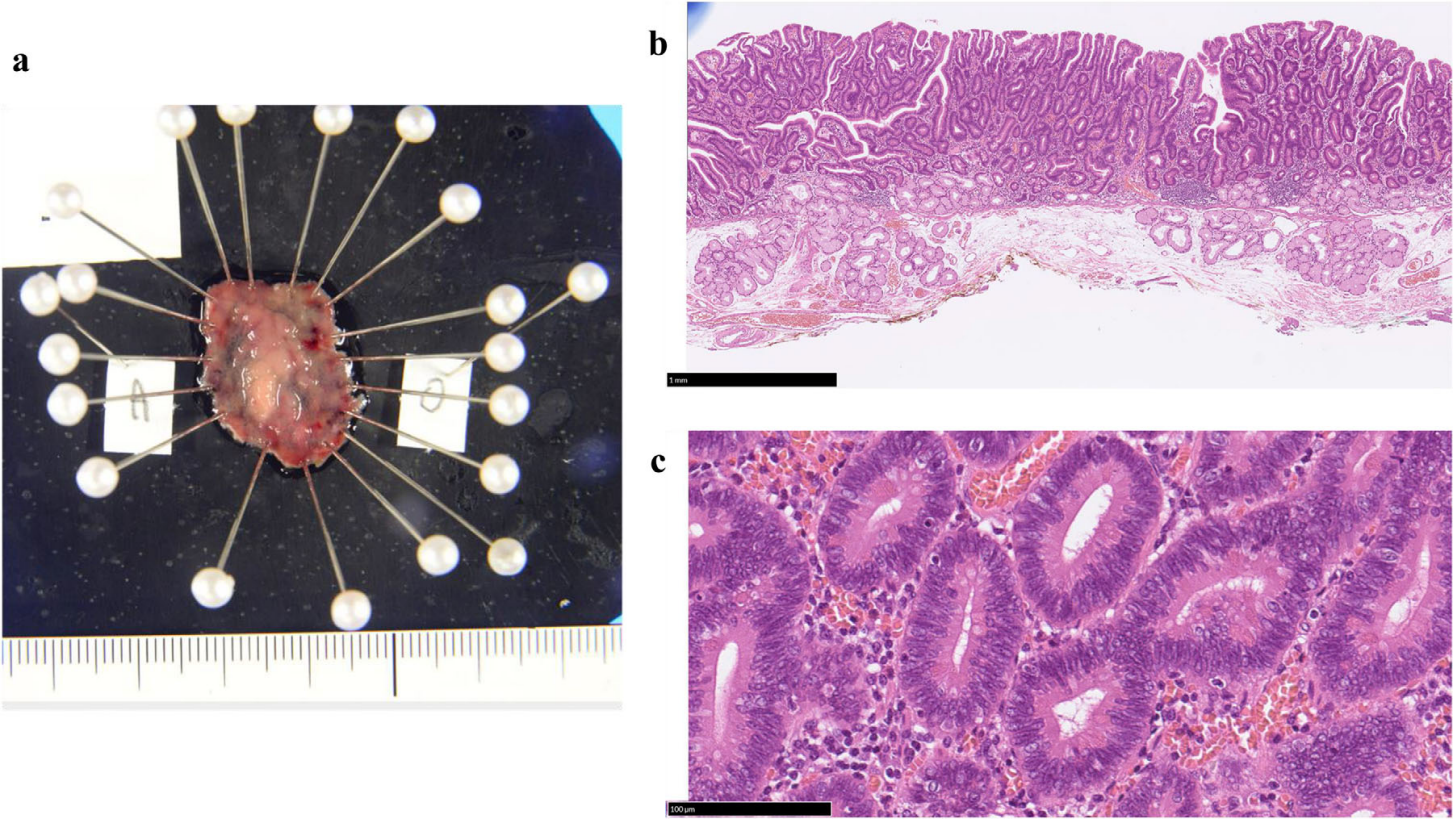
Macroscopic and histopathological findings. (a) The resected specimen measured 20 × 12 mm and was classified as type 0‐IIa. (b) Low‐power histopathological view showing a tubular adenomatous lesion confined to the mucosal layer without submucosal invasion (H&E stain; scale bar = 1 mm). (c) High‐power histopathological view demonstrating dense tubular proliferation of atypical epithelial cells with eosinophilic cytoplasm and enlarged, elongated nuclei confined to the mucosal layer (H&E stain; scale bar = 100 µm). The lesion was diagnosed as a low‐grade intestinal‐type duodenal adenoma. Both horizontal and vertical margins were negative, and no lymphovascular invasion was identified.

The resected specimen measured 20 × 12 mm and was classified as type 0‐IIa (Figure 4a). Histopathology confirmed a low‐grade intestinal‐type duodenal adenoma with negative horizontal and vertical margins and no vascular invasion (Figure 4b,c).

## 3 Discussion

D‐LECS is a minimally invasive procedure combining ESD with laparoscopic reinforcement and has been reported as a safe and effective treatment for duodenal tumors. In the duodenum, ESD alone is associated with a high risk of perforation and delayed complications, particularly when lesions are large or located in anatomically complex regions [2, 3]. D‐LECS was developed to overcome these limitations by combining endoscopic resection with laparoscopic reinforcement to reduce the risk of leakage and perforation [5, 6]. This approach preserves the organ and may avoid pancreaticoduodenectomy [4].

The lesion measured only 10 mm, but its close proximity to the papilla of Vater (approximately 3 mm) made safe snaring with adequate margins technically difficult. Accordingly, ESD was selected to achieve en bloc resection while minimizing the risk of papillary injury and preserving papillary function.

Previous reports suggest that D‐LECS is indicated for lesions located at a sufficient distance from the papilla of Vater [6]. Although the lesion was closer to the papilla than in previous reports, pancreaticoduodenectomy was considered excessively invasive because the lesion was considered benign preoperatively. D‐LECS was therefore selected as an organ‐preserving approach despite the challenging location.

Because complete laparoscopic reinforcement is anatomically difficult for periampullary lesions on the pancreatic side of the duodenum [7], the principal role of D‐LECS was not complete serosal reinforcement. Rather, D‐LECS improved endoscopic maneuverability through Kocher mobilization, while allowing intraperitoneal assessment, additional reinforcement, drainage, and retroperitoneal fixation. Kocher mobilization created a more linear endoscopic approach with improved torque transmission and scope stability, facilitating safe ESD and defect closure. Thus, D‐LECS is a hybrid approach that facilitates safe endoscopic treatment of technically demanding periampullary lesions.

However, lesions near the papilla of Vater require meticulous defect closure because thermal injury or postoperative edema may impair pancreaticobiliary outflow. Post‐ESD ulcers in conventional D‐LECS are routinely reinforced using full‐thickness horizontal mattress sutures. In this case, full‐thickness suturing was considered to carry a risk of papillary deformation because of the lesion's close proximity to the papilla. ROLM was therefore selected for precise mucosal closure under endoscopic visualization, followed by supplementary laparoscopic seromuscular suturing to reinforce the defect and reduce the risk of delayed perforation. Although only 10 mm in size, an adequate resection margin resulted in a relatively large post‐ESD mucosal defect. Accordingly, complete mucosal closure using ROLM was considered necessary. Endoscopic reassessment confirmed closure without duodenal stenosis or papillary deformation.

ENPD and ENBD are widely used to prevent pancreatitis and cholangitis after endoscopic biliary and pancreatic procedures by decompressing the pancreatic and biliary ducts [8]. Given its proximity to the papilla, thermal injury and postoperative edema after ESD were considered likely to impair pancreaticobiliary outflow, potentially causing pancreatitis or cholangitis. Prophylactic pancreaticobiliary drainage was therefore performed. Another rationale for combining ENPD and ENBD was to prevent mixing of pancreatic juice and bile around the post‐ESD defect. Mixed pancreaticobiliary fluid has strong digestive activity, and leakage outside the intestinal tract may cause severe autodigestive tissue injury, including injury to adjacent major vessels. Bacteria in bile may also exacerbate tissue injury. Simultaneous external drainage was therefore considered necessary to reduce digestive and infectious stress on the post‐ESD closure site. ENPD and ENBD require selective pancreaticobiliary cannulation and may cause ERCP‐related adverse events, including pancreatitis and cholangitis [9]. Accordingly, these procedures should not be used routinely but should be reserved for lesions near the papilla of Vater when transient pancreaticobiliary obstruction is anticipated. Kobayashi et al. performed prophylactic pancreatic duct stenting before ESD to facilitate safe dissection and avoid pancreatic duct obstruction during defect closure [10]. In contrast, ENPD and ENBD were placed after completion of D‐LECS to avoid interference with ESD and defect closure. Selective pancreaticobiliary cannulation remained feasible after D‐LECS, ROLM closure, and laparoscopic seromuscular suturing, suggesting that these procedures did not preclude subsequent papillary access.

However, evidence supporting prophylactic ENPD and ENBD remains limited because reports of D‐LECS for periampullary lesions are scarce. In this case, the procedure was completed without complications, suggesting that combined ENPD and ENBD may be a useful adjunct to D‐LECS for selected periampullary lesions while maintaining minimal invasiveness.

## Author Contributions

**Keisuke Manaka**: conceptualization, methodology, investigation, validation, visualization, project administration, and writing – original draft. **Hiroki Harada**: conceptualization, methodology, validation, supervision, visualization, project administration, and writing – review and editing. **Takuya Wada**: methodology, investigation, and validation. **Gen Kitahara**: methodology, investigation, and validation. **Kosuke Okuwaki**: methodology, investigation, validation, and project administration. **Kota Okuno**: methodology and investigation. **Shohei Fujita**: methodology and investigation. **Tadashi Higuchi**: methodology and investigation. **Koshi Kumagai**: methodology, validation, and project administration. **Naoki Hiki**: methodology, supervision, project administration, and writing – review and editing.

## Conflicts of Interest

The authors declare no conflicts of interest.

## Funding

The authors have nothing to report.

## Supporting information




**FILE S1 Detailed procedural time**: detailed breakdown of the procedural time for each operative component.

**VIDEO S1** Surgical procedure of D‐LECS for a periampullary duodenal adenoma. The video demonstrates mobilization and straightening of the duodenum using the Kocher maneuver, ESD of the tumor adjacent to the papilla of Vater, closure of the mucosal defect using the ROLM, reinforcement with laparoscopic seromuscular suturing, retroperitoneal fixation of the duodenum, and drain placement.
