# Supplementary material for: Duodenal Laparoscopy and Endoscopy Cooperative Surgery Combined With Endoscopic Nasopancreatic Drainage and Endoscopic Nasobiliary Drainage for a Duodenal Adenoma Near the Papilla of Vater: A Case Report
Source: DEN Open. 2026 Sep 3;7(1):e70390. doi: 10.1002/deo2.70390 (PMC13540642; doi:10.1002/deo2.70390)
Supplement: Supplementary file 1 [file DEO2-7-e70390-s002.docx]

**Supporting Information**

**Supporting Information S1. Detailed procedural time.**

The total procedural time was 356 minutes. The active procedural components consisted of laparoscopic preparation (44 min), Kocher mobilization (18 min), endoscopic submucosal dissection (47 min), ROLM closure (35 min), laparoscopic seromuscular suturing (21 min), and ERCP with ENPD and ENBD placement (34 min). The remaining time included patient transfer to an operating room equipped with fluoroscopy, fluoroscopic setup, re-laparoscopic observation after ERCP, retroperitoneal fixation of the duodenum, drain placement, wound closure, and anesthesia-related procedural intervals.
